# Supplementary material for: SARS-CoV-2 Vaccination and Clinical Presentation of COVID-19 in Patients Hospitalized during the Delta- and Omicron-Predominant Periods
Source: J Clin Med. 2023 Jan 26;12(3):961. doi: 10.3390/jcm12030961 (PMC9918275; doi:10.3390/jcm12030961)
Supplement: Supplementary file 1 [file jcm-12-00961-s001.zip › jcm-2168504-supplementary.pdf]

Supplementary Table S1. Categorization of underlying chronic illnesses.

Chronic Lung Disease	• Asthma<br>• Cystic fibrosis<br>• Chronic obstructive pulmonary disease<br>• Obstructive sleep apnea<br>• Oxygen dependence<br>• Pulmonary fibrosis<br>• Restrictive lung disease<br>• Sarcoidosis
Chronic Metabolic Disease	• Adrenal disorders<br>• Diabetes mellitus<br>• Glycogen or other storage diseases<br>• Hyper/Hypopituitary function<br>• Hyper/Hypoparathyroidism<br>• Congenital metabolic diseases<br>• Metabolic Syndrome<br>• Thyroid dysfunction
Hematologic Conditions	• Chronic anemia<br>• Coagulopathy or other bleeding disorder<br>• Myelodysplastic syndrome<br>• Polycythemia vera<br>• Sickle cell disease<br>• Thalassemia<br>• Thrombocytopenia
Cardiovascular Disease	• Atherosclerotic cardiovascular disease<br>• Cardiac arrhythmias<br>• Cardiomyopathy<br>• Congenital heart disease<br>• History of aortic aneurysm<br>• History of transient ischemic attack/Stroke<br>• Heart failure<br>• Heart valve disease<br>• Hypertension<br>• Peripheral artery disease<br>• Pulmonary hypertension
Neurologic Disorders	• Amyotrophic lateral sclerosis<br>• Cerebral palsy<br>• Cognitive dysfunction<br>• Dementia/Alzheimer's disease<br>• Developmental delay<br>• Epilepsy<br>• Multiple sclerosis<br>• Muscular dystrophy<br>• Myasthenia gravis<br>• Parkinson's disease<br>• History of brain injury<br>• History of Guillain-Barre syndrome<br>• Paraplegia/Quadriplegia
Immunocompromising Conditions	• Active treatment with immunosuppressive medications<br>• Complement deficiency<br>• Hematologic cancer [1]<br>• History of hematopoietic stem cell transplant within 2 years<br>• History of solid organ transplant<br>• History of splenectomy<br>• HIV infection<br>• Immunoglobulin deficiency

	• Solid organ malignancy [1]
Renal Disease	• Chronic kidney disease<br>• Dialysis<br>• End stage renal disease<br>• Glomerulonephritis<br>• Nephrotic syndrome<br>• Polycystic kidney disease
Gastrointestinal/Liver Disease	• Alcoholic hepatitis<br>• Autoimmune hepatitis<br>• Barrett's esophagitis<br>• Chronic liver disease<br>• Chronic pancreatitis<br>• Cirrhosis/End stage liver disease<br>• Crohn's disease<br>• Esophageal varices<br>• Hepatitis B, chronic<br>• Hepatitis C, chronic<br>• Non-alcoholic fatty liver disease<br>• Ulcerative colitis
Rheumatologic/Autoimmune Conditions	• Ankylosing spondylitis<br>• Dermatomyositis<br>• Juvenile idiopathic arthritis<br>• Microscopic polyangiitis<br>• Polyarteritis nodosum<br>• Polymyalgia rheumatica<br>• Polymyositis<br>• Psoriatic arthritis<br>• Rheumatoid arthritis<br>• Systemic Lupus Erythematosus<br>• Temporal/Giant Cell arteritis<br>• Vasculitis, other
Other	• Other underlying medical conditions not specified above

[1] Currently treated or diagnosed in the past 6 months

Supplementary Table S2. Characteristics of patients hospitalized for COVID-19 by vaccination status.

**Characteristic**	**Fully vaccinated** [1] ***n* = 334/929 (36%)**	**Unvaccinated** ***n* = 595/929 (64%)**	***p*-Value** [2]
Age	76 (67–84)	65 (53–78)	**<0.001**
Men	197 (59.0)	314 (52.8)	0.074
Charlson index	4 (3–6)	3 (1–4)	**<0.001**
Chronic illnesses [3]			
-Cardiovascular disease	251 (75.1)	322 (54.1)	**<0.001**
-Pulmonary disease	66 (19.8)	71 (11.9)	**0.001**
-Asthma	22 (6.6)	34 (5.7)	0.667
-COPD	39 (11.7)	29 (4.9)	**<0.001**
-Diabetes type II	110 (32.9)	103 (17.3)	**<0.001**
-Obesity (body mass index ≥ 30)	103 (30.8)	197 (33.1)	**<0.001**
-Immunocompromising condition	57 (17.1)	31 (5.2)	**<0.001**
One or more comorbidities	300 (89.8)	104 (17.5)	**<0.001**
One comorbidity	35 (10.5)	391 (65.7)	**<0.001**
Resident of long-term care facility	45 (13.5)	16 (2.7)	**<0.001**
Received booster dose	56 (16.8)	0 (0)	**<0.001**
Self-reported previous infection	4 (1.2)	4 (0.7)	0.468
Primary vaccine received			
-BNT162b2	252 (75.4)	—	—
-ChAdOx-1S	38 (11.4)	—	—
-Ad.26.COV2.S	24 (7.2)	—	—
-mRNA-1273	20 (6.0)	—	—
Therapy			
-Remdesivir	57 (17.1)	65 (10.9)	**0.009**
-Corticosteroids	290 (86.8)	540 (90.8)	0.076
-Dexamethasone	253 (75.7)	412 (69.2)	0.041
-Methylprednisolone	91 (27.2)	226 (38.0)	**0.001**
-Tocilizumab	0 (0)	16 (2.7)	**0.002**
-Monoclonal antibodies	11 (3.3)	21 (3.5)	1.000
Hypoxemic at admission	280 (83.8)	527 (88.6)	0.044
Critically severe disease (WHO score 7–10) [4]	75 (22.5)	154 (25.9)	—
Death (WHO score 10) [4]	59 (17.7)	91 (15.3)	—

COPD: Chronic obstructive pulmonary disease. Data are *n* (%) or median (95% interquartile range). [1] Received the second dose of BNT162b2, mRNA-1273 or ChAdOx-1S or first dose of Ad.26.COV2.S at least 14 days before symptom onset. [2] Due to multiple comparisons, *p*-value < 0.01 was considered significant (marked in bold). [3] See Supplementary Table S1 for definitions of chronic illnesses. [4] Assessed using the World Health Organization COVID-19 Clinical Progression Scale [21].

Supplementary Table S3. Association between time since the last vaccine dose and progression to critically severe disease (the World Health Organization COVID-19 Clinical Progression Scale 7–10) [21].

**Characteristics**	**Odds ratio (95% CI)**	***P* value** [1]
Intercept	0.02 (0.00–0.20)	**< 0.001**
Time since last vaccination in weeks [2]	0.99 (0.96–1.01)	0.393
SARS-CoV-2 variant (Omicron vs. Delta)	1.33 (0.76–2.32)	0.316
Age	1.03 (1.00–1.06)	0.082
Sex (male vs. female)	1.07 (0.61–1.86)	0.816
Charlson comorbidity index	1.09 (0.96–1.25)	0.196
Immunocompromising condition present (yes vs. no)	1.67 (0.77–3.60)	0.193

CI, confidence interval

[1] *P* value < 0.05 was considered significant (marked in bold). [2] Using the date of the booster dose for 56 patients who received it.

Supplementary Table S4. Association between SARS-CoV-2 antibody titer and progression to critically severe disease (the World Health Organization COVID-19 Clinical Progression Scale 7–10) [21].

**Characteristics**	**Odds ratio (95% CI)**	***P* value [1]**
Intercept	0.02 (0.00–0.32)	**0.005**
SARS-CoV-2 antibody titer	1.00 (1.00–1.00)	0.593
SARS-CoV-2 variant (Omicron vs. Delta)	0.99 (0.50–1.94)	0.971
Age	1.02 (0.99–1.06)	0.222
Sex (male vs. female)	1.36 (0.68–2.71)	0.384
Charlson comorbidity index	1.09 (0.92–1.30)	0.321
Immunocompromising condition present (yes vs. no)	1.10 (0.42–2.89)	0.849

CI, confidence interval

[1] *P* value < 0.05 was considered significant (marked in bold).
